# Gonadal transcriptome analysis of hybrid triploid loaches (*Misgurnus anguillicaudatus*) and their diploid and tetraploid parents

**DOI:** 10.1371/journal.pone.0198179

**Published:** 2018-05-24

**Authors:** He Zhou, Qi-Zheng Xu, Rui Zhang, Zi-Xin Zhuang, Yin-Qiang Ma, Wei Wang, Tian-Yu Ma, Yi Sui, Yang Liu, Xiaojuan Cao

**Affiliations:** 1 Key Laboratory of Mariculture and Stock Enhancement in North China’s Sea, Ministry of Agriculture, Dalian Ocean University, Dalian, China; 2 Dalian Medical University, Dalian, China; 3 Ma'anshan Municipal Agriculture Commission, Anhui, China; 4 College of Fisheries, Key Lab of Agricultural Animal Genetics, Breeding and Reproduction of Ministry of Education/Key Lab of Freshwater Animal Breeding, Ministry of Agriculture, Huazhong Agricultural University, Wuhan, Hubei, China; Ohio State University, UNITED STATES

## Abstract

Hybrid triploid loaches (*Misgurnus anguillicaudatus*) were generated from natural tetraploid and diploid loaches. We studied the gonads of parents and offspring from direct and reciprocal crosses through histological and transcriptome analyses. The triploid offspring had inferior ability to form sperm and egg cells compared with diploid forms. After sequencing the transcriptomes, 655,109,158 clean reads were obtained, and 62,821 unigenes and 178,962 transcripts were assembled. Of these unigenes, 23,189 were annotated in the GO database, 18,525 in the KEGG database and 24,661 in the KOG database. 36 fertility-related genes were found to be differentially expressed between the direct cross (2n × 4n) progenies and their parents, while 53 fertility-related genes between the reciprocal cross (4n × 2n) progenies and their parents. Following protein-protein interaction network analyses, 54 differentially expressed genes, including *PLCB4*, *cyp17a1* and *Pla2g4d*, were mined, yielding candidate genes involved in the poor fertility of hybrid triploid loaches. This is the first report of transcriptomes of gonads of hybrid triploid loaches and their parents, offering a substantial contribution to sequence resources for this species and providing a deep insight into the molecular mechanism controlling the fertility of hybrid triploid fish.

## Introduction

Loach (*Misgurnus anguillicaudatus*; Cypriniformes; Cobitidae) is one of the endemic fishes of Asia and a common small freshwater fish in China. The loach is distributed in all parts of China. For delicious taste and traditional Chinese medicine value, it has become one of the most important cultured fish species in China. The loach exhibits a range of polyploidy: in addition to diploid, there are natural triploid [1], tetraploid [2–5] and hexaploid [3] forms in China. This ploidy has received close attention by scholars internationally. Thus, Arai et al. [4,6–8] reported the distribution of natural triploid loach in Japan, but found no natural tetraploid forms. The tetraploid variety has the characteristics of fast growth, low oxygen consumption and high nutritional value. It has important germplasm value in genetics and breeding, and as such is a unique and valuable resource in China. The genome formation of natural tetraploid loach in China was studied systematically by Li [5,9–14] on cellular and molecular levels. These showed that the natural tetraploid loach is a genetic form (4n = 100) that produces normal 2n eggs and 2n spermatozoa. A new hybrid intraspecific triploid was prepared by crossing diploid and tetraploid loaches [9], in combination with cross-breeding and triploid breeding. In theory, triploid fish are expected to be sterile, but a few individuals can reach sexual maturity and produce small amounts of eggs and spermatozoa. Therefore, we have previously studied the chromosome numbers, DNA content, meiosis and reproductive characteristics of triploid loaches [15,16] with fertility as the endpoint. From the viewpoint of epigenetics, differences in DNA methylation levels and the control of key genes in hybrid triploid loaches and their parents have been analyzed [17]. However, the molecular mechanisms controlling the fertility of these fish remain unclear.

Transcriptomics can be used to evaluate gene structure and function at the whole organism level, and to reveal the molecular mechanism of specific biological processes [18]. The transcriptomal high-throughput sequencing technique has been applied successfully to multiple model and natural organisms. Thus, the Sanger Research Institute has published the zebrafish genome reference sequence [19]. Yuan [20] used the Illumina HiSeq technique to study the gene expression of gonads during different developmental stages of the Nile tilapia (*Oreochromis niloticus*). Zhang et al. [21] identified the innate immune genes of carp *(Cyprinus carpio)* using the RNA sequencing (RNA-seq) approach, and found 39 transcripts, isoforms and genes encoding a Toll/interleukin-1 receptor (TIR) structure. Luo et al. [22] reported the ovarian transcriptome of diploid and tetraploid loaches obtained using an RNA-seq approach. The liver transcriptome of male and female zebrafish (*Danio rerio*) was studied by high-throughput RNA-serial analysis of gene expression (SAGE) sequencing, and the molecular basis of sex differences in the liver of male and female fish was explored by Zheng et al. [23]. Here, a new hybridized triploid loach was generated using natural tetraploid and diploid loaches, and the mature gonads of parents and offspring of the direct and reciprocal crosses were used for histological and transcriptome studies. The molecular mechanism of the fertility of hybrid triploid loaches was investigated in terms of transcriptomes, which will provide reference values for the commercial application of this fish.

## Materials and methods

### Ethics statement

This study was performed according to the Guide for the Care and Use of Laboratory Animals in Dalian Ocean University, Dalian, China. All animal experiments were approved by the animal study ethical committee of Dalian Ocean University, and complied with Chinese laws, regulations and ethics.

### Materials

Natural diploid and tetraploid loaches were collected from Wuhan City, Hubei Province, P. R. China. All were kept in aquaria (25 ± 1°C) in the laboratory of Dalian Ocean University.

### Ploidy identified by flow cytometry

Diploid loaches were used as the control group. Different ploidy male and female individuals were used to recover tissues clipped from the tail. These were placed in a centrifuge tube containing lysate until the tissue mass disappeared, and DAPI (4',6-diamidino-2-phenylindole) was added to each tube. Tests of ploidy were performed using flow cytometry (Partec PAS-III, Partec, Munster, Germany).

### Artificially induced spawning and insemination

The parents were chosen from well-developed diploid and natural tetraploid loaches injected with human chorionic gonadotropin (HCG) (injection dose for females 20–25 IU·g^−1^; males 10–12.5 IU·g^−1^). After 12 h, the abdomens of female fishes were pressed gently to discharge the eggs into a 9 cm culture dish. Semen from male fishes was similarly extruded from genital pores on both sides of the body. It was collected in centrifuge tubes and diluted 100 times with Kurokura solution (750 mg NaCl, 20 mg CaCl_2_, 20 mg NaHCO_3_, and 20 mg KCl dissolved in 100 ml distilled water). Using dry fertilization, the hybrid combinations namely a direct cross (2n × 4n) and a reciprocal cross (4n × 2n) were obtained. During the incubation periods, the temperature was maintained at 25 ± 1°C. The fresh water was aerated, and any dead fry were removed immediately from the nursery aquarium.

### Sample collection

After the experimental loaches had reached sexual maturation, the method of euthanasia (i.e. decapitation) was used for loaches anesthetized with 100 mg/L tricaine methanesulfonate (MS-222). And then the loaches were placed on ice for tissues collections. The gonads of four parents and eight offspring were removed. Parts were fixed in Bouin’s solution and stored in 80% alcohol for 24 h before being prepared for histology of gonadal tissue sections. The other parts were placed in liquid nitrogen for temporary storage, and then stored in tubes in a –80°C freezer before RNA extraction.

### Preparation and observation of gonadal tissue sections

Fixed samples were dehydrated in an alcohol gradient, cleared in xylene, embedded in paraffin wax, then serially sectioned using a microtome (Leica Instruments, Nussloch, Germany) at 6–8 μm thickness. Dewaxed sections were stained with hematoxylin and eosin (HE) and sealed on slides under neutral gum. Images were taken using an Olympus AH2 camera (Olympus, Tokyo, Japan).

### Library construction and sequencing

RNA extraction used enrichment with magnetic beads coated with oligo (dT) primer. The extracted mRNAs were broken randomly into short fragments using fragmentation buffer, and the first strand cDNA was synthesized as random hexamers using a fragment of mRNA as a template. Subsequently, buffer, dNTPs, RNase H and DNA polymerase I were added to synthesize the second strand cDNA. Then we used AMPure XP beads to double-purify products, T4 DNA polymerase and Klenow DNA polymerase to fix the sticky end of the DNA to the flat end. 3′-end added base A and the joint, then used AMPure XP beads (Agencourt Bioscience, Beverly, MA, USA) for fragment selection. Finally, qPCR amplification was used to obtain the final sequencing library. Sequencing was done by LC-Bio Technologies Co., Ltd. (Hangzhou, P. R. China) (http://www.lc-bio.com) using an Illumina HiSeq2000/2500 machine (Illumina, San Diego, CA, USA) for paired-end sequencing.

### *De novo* assembly and functional annotation

Valid sequencing data were obtained by removing any linker sequence containing an uncertain information base (N) ratio greater than 5% reads, any low quality of the reads (Q ≤ 10, the base number accounted for 20% of the entire read). The primary sequencing amounts, effective sequencing amounts, Q20, Q30 and GC contents were recorded. The loach has no reference genome, so sequence data were spliced using Trinity software (https://github.com/trinityrnaseq/trinityrnaseq/wiki) to obtain sequences of genes and transcripts. Functional annotation of sequences was carried out by LC-Bio Technologies Co., Ltd., based on the following databases: SWISS-PROT, a manually annotated and reviewed protein sequence database (ftp://ftp.uniprot.org/pub/databases/uniprot/currentrelease/knowledgebase/complete/uniprotsprot.fasta.gz); NR, NCBI non-redundant protein sequences (ftp://ftp.ncbi.nlm.nih.gov/blast/db/FASTA/nr.gz); KEGG, Kyoto Encyclopedia of Genes and Genomes (http://www.kegg.jp/kegg/download/); KOG, EuKaryotic Ortholog Groups (http://www.ncbi.nlm.nih.gov/COG/grace/shokog.cgi); and Pfam, a widely used protein family and structure domain database (ftp://ftp.sanger.ac.uk/pub/databases/Pfam/releases/Pfam27.0/Pfam-A.fasta.gz). All searches were conducted with Blast (http://blast.ncbi.nlm.nih.gov/) using a minimum e-value of ≤ 1e^–5^ as a threshold.

### Identification of differentially expressed genes (DEGs)

Gene expression level was normalized against the Reads Per Kilobase of transcripts per Million mapped reads (RPKM) value, which considers the effect of sequencing depth and gene length for the read count at the same time, and is currently the most commonly used method for estimating gene expression levels. DEGs and their corresponding P values were determined using the methods described by Audic [24] in 1997, and the significance threshold of the P value in multiple tests was set based on the false discovery rate (FDR). The fold changes (log_2_ RERPKM/PERPKM) were also estimated according to the normalized gene expression level. For this study, “FDR< 0.001 and |log_2_ fold change|> 2” were used as the thresholds to define DEGs. The protein-protein interaction network analyses of fertility-related DEGs were performed and the key genes causing the poor fertility of hybrid triploid loach were obtained.

### Validation of RNA-seq results by qPCR

To test the reliability of the RNA-Seq results, DEGs involved in the development of gonads tissues were selected for validation using qPCR. The first strand synthesis of cDNA was carried out using Revert Aid Premium Reverse Transcriptase kits (# EP0733; Thermo Scientific, Waltham, MA, USA). The reactions were performed in an ABI Step One Plus machine (Thermo Scientific). The cDNA samples diluted 10 times served as test templates. The reaction mix included SYBR Green qPCR Master Mix 10 μl, 0.4 μl fragment F (10 μM), 0.4 μl fragment R (10 μM), 7.2 μl H_2_O and 2 μl template cDNA. The following thermal cycling parameters were used: initiation at 95°C for 3 min followed by 45 cycles of 95°C for 7s for denaturation, 57°C for 10s for annealing, and 72°C for 15 s for extension. All experiments were conducted with three biological replicates for each sample. The relative expression levels were normalized to the endogenous housekeeping gene β-actin and expression ratios were calculated using the 2^−ΔΔCt^ [25] method.

## Results

### Ploidy identification

**Fig 1:**
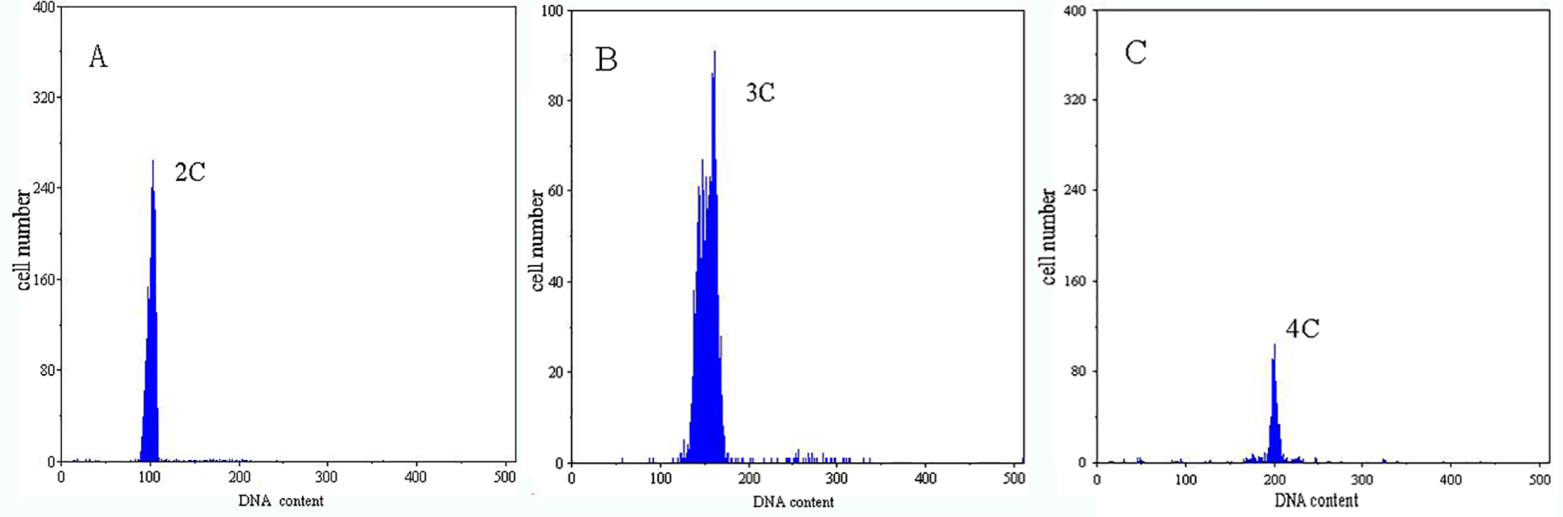
**DNA-content flow-cytometrical histograms of diploid (A), triploid (B), and tetraploid (C) loach *Misgurnus anguillicaudatus***.

Flow cytometry analysis indicated that the diploid, triploid and tetraploid cell population DNA contents were 2C DNA (Fig 1A), 3C DNA (Fig 1B) and 4C DNA (Fig 1C), respectively. The DNA content ratio in individual blood cells of diploid, triploid and tetraploid forms was 1:1.5:2.

### Histology and comparison of mature gonads

**Fig 2:**
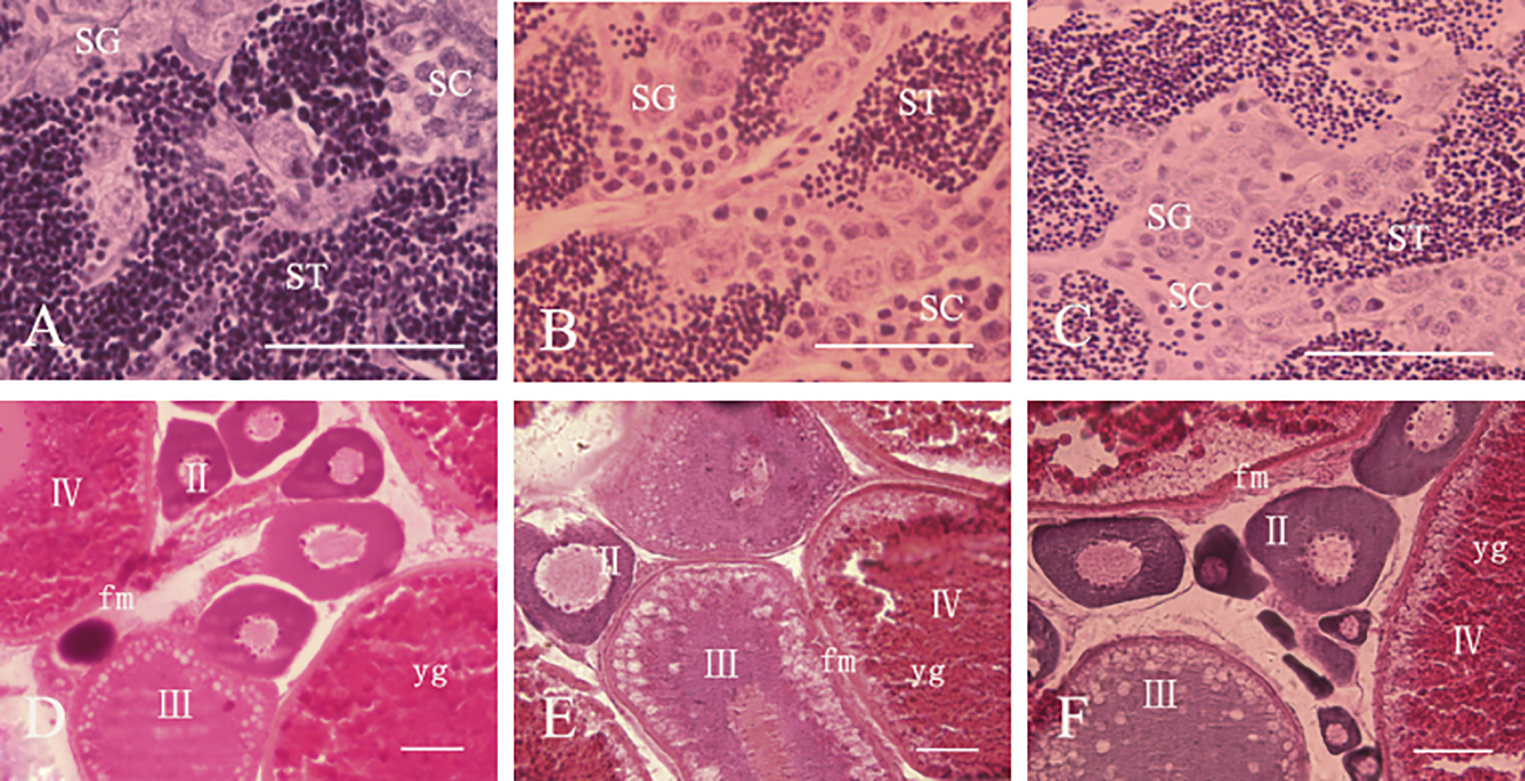
Micrographs of the testes and ovaries of hybrid triploid loach. A: The testis of diploid; B: The testis of hybrid triploid (2n×4n); C: The testis of hybrid triploid (4n×2n); D: The ovary of diploid; E: The ovary of hybrid triploid (2n×4n); F: The ovary of hybrid triploid (4n×2n); SG: spermatogonia; SC: spermatocyte; ST: spermatid; Ⅱ: stage Ⅱ oocyte; Ⅲ: stage Ⅲ oocyte; Ⅳ: stage Ⅳ oocyte; yg: yolk granule; fm: follicle membrane; Bars = 50μm.

Spermatogonia (SG), spermatocytes (SC), and large numbers of mature spermatid (ST) were observed in diploid and hybrid triploid testes, but the mature sperm content was lower than in normal diploid forms (Fig 2A, 2B and 2C). Thus the male hybrid triploid can produce spermatozoa, but the output is less than in the control diploid male testis. Diploid and hybrid triploid female ovaries also contained second, third and fourth phase oocytes, yolk granules (yg) and follicular membranes (fm) (Fig 2D, 2E and 2F).

### Sequence assembly and unigene annotation

Preliminary analysis showed that the 12 samples (Table 1) used in the trial experiment had optical densities (OD) at 260/280 wavelength of >1.8, indicating no protein contamination. The RNA integrity numbers (RINs) were 8.1–9.3. The results showed that the RNAs were free of protein contamination and their integrities were high and met the requirements of sequencing. The detailed test result report is listed in S1 Table.

**Table 1:** Sample information.

Sample No.	Sample Type
**PF(2n×4n)**	2n female parent
**PM(2n×4n)**	4n male parent
**OF(2n×4n)-1**	F_1_♀(2n×4n) -1
**OF(2n×4n)-2**	F_1_♀(2n×4n) -2
**OM(2n×4n)-1**	F_1_♂(2n×4n) -1
**OM(2n×4n)-2**	F_1_♂(2n×4n) -2
**PF(4n×2n)**	4n female parent
**PM(4n×2n)**	2n male parent
**OF(4n×2n)-1**	F_1_♀(4n×2n) -1
**OF(4n×2n)-2**	F_1_♀(4n×2n) -2
**OM(4n×2n)-1**	F_1_♂(4n×2n) -1
**OM(4n×2n)-2**	F_1_♂(4n×2n) -2

Illumina-based RNA-Seq analysis was conducted with gonadal tissue samples from hybrid triploid loaches and their parents. A total of 661.34 million raw data sequences with an average length of 100 bp were generated. After trimming out low-quality reads and short read sequences, a total of 655.11 million sequences of clean data (99.05%) were obtained. The average GC content was 46.46% (S2 Table), and these reads were used for the following analysis of 178,962 transcripts and 62,821 unigenes. The N50 values of transcripts and unigenes were 2,149 and 2,121, respectively. A summary of the assembly data is given in Table 2. The size distributions of contigs, transcripts and unigenes are shown in S1 Fig and S2 Fig.

**Table 2:** Length of gene and transcript.

Length	Gene		transcript	
	Count	Percentage	Count	Percentage
200–300	24530	39.05	45523	25.44
400–500	8229	13.1	22274	12.44
600–1000	9593	15.27	34273	19.15
1100–1900	9379	14.92	36948	20.64
>2000	11090	17.65	39944	22.32
**Total Number**	62821		178962	
**Total Assembled bases**	69673053		235205375	
**N50 Length**	2121		2149	
**Mean Length**	1109		1314	
**Median Length**	557		888	
**Mean GC%**	42.00		41.47	
**Median GC%**	42.40		41.80	

**Fig 3:**
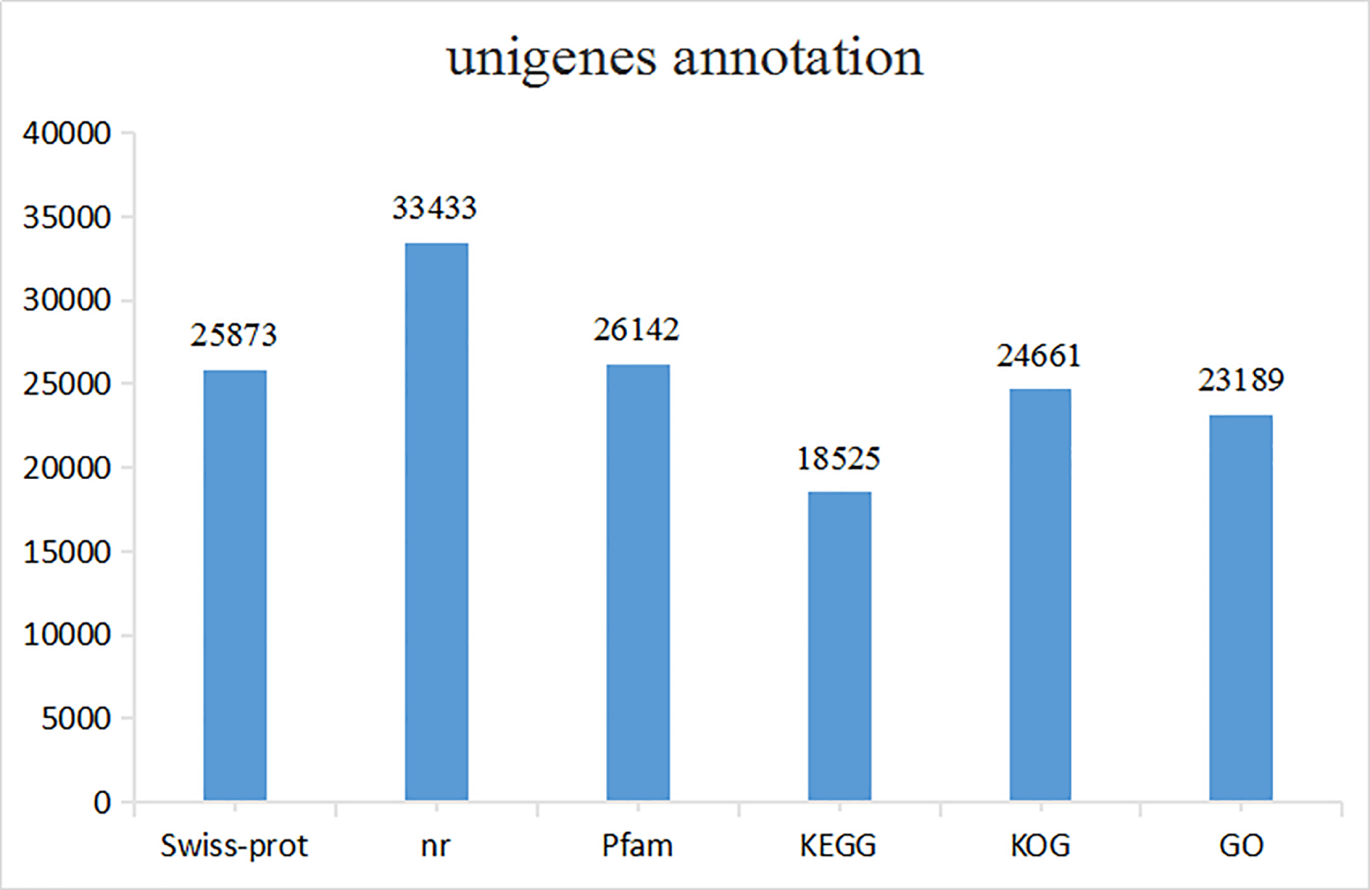
Annotation of unigenes in different databases.

In all, 62,821 unigenes were annotated by Blastx and Blastn against six public databases (Swiss-Prot, NR, Pfam, KEGG, KOG, and GO), with a threshold of 10^−5^. According to the numbers and proportions of unigenes in the different databases, there were differences in the numbers of genes annotated successfully, because these databases use different filter conditions, such as protein sequence, protein topology, and the metabolic pathways involved. The NR database annotated 33,433 unigenes successfully, accounting for 53.22% of the total number of genes. The number of genes was least well covered in the KEGG database where 18,525 unigenes were annotated, accounting for 29.05% of the total (Fig 3).

### Unigene classification

**Fig 4:**
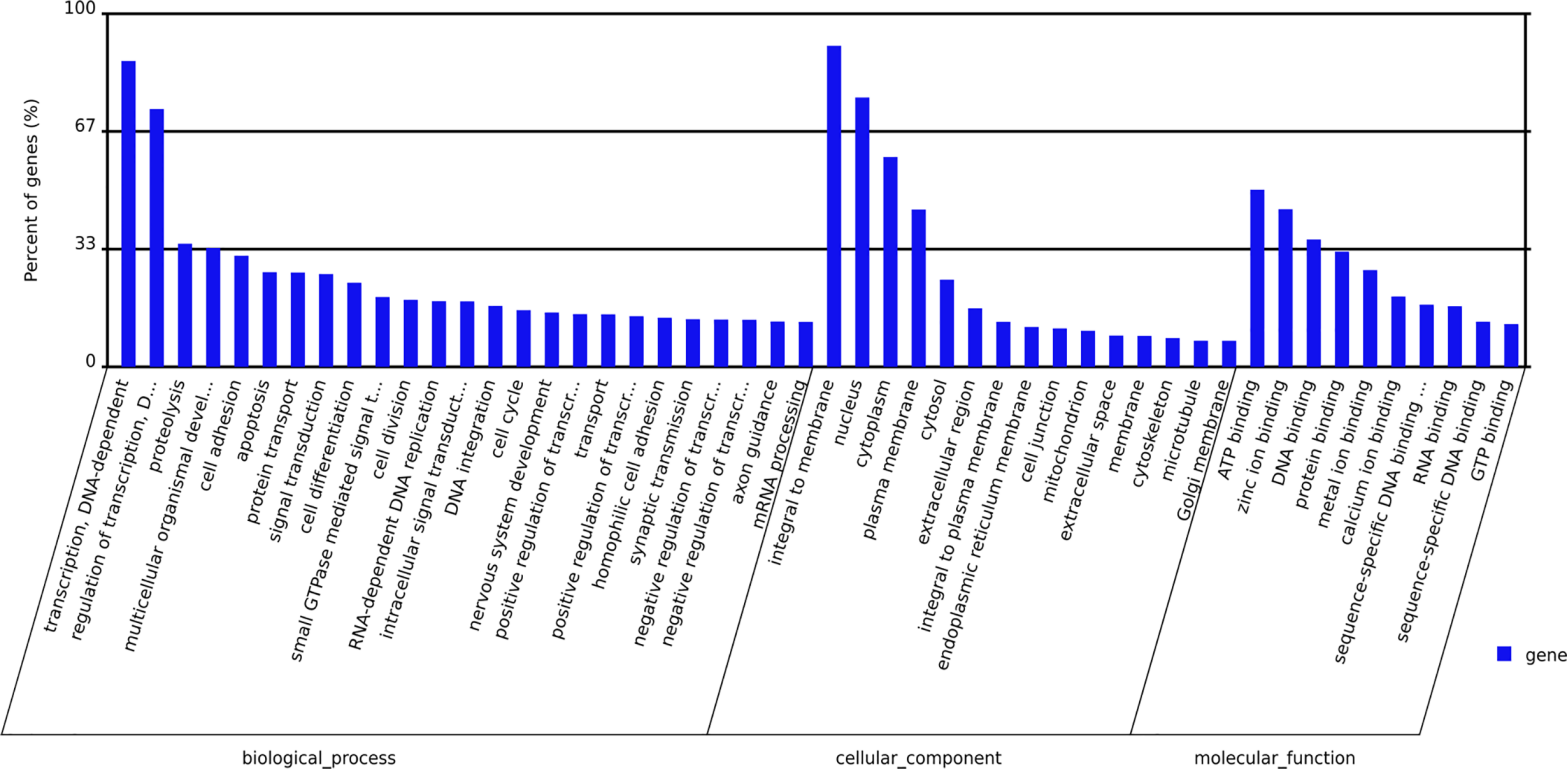
GO classification of unigenes.

In all, 23,189 annotated genes were obtained by Gene Ontogeny (GO) annotation, accounting for 36.91% of the total number of unigenes. GO analysis identified three main functional pathways: biological pathways, cellular components and molecular functions. These were subdivided into 52 functional categories (Fig 4).

**Fig 5:**
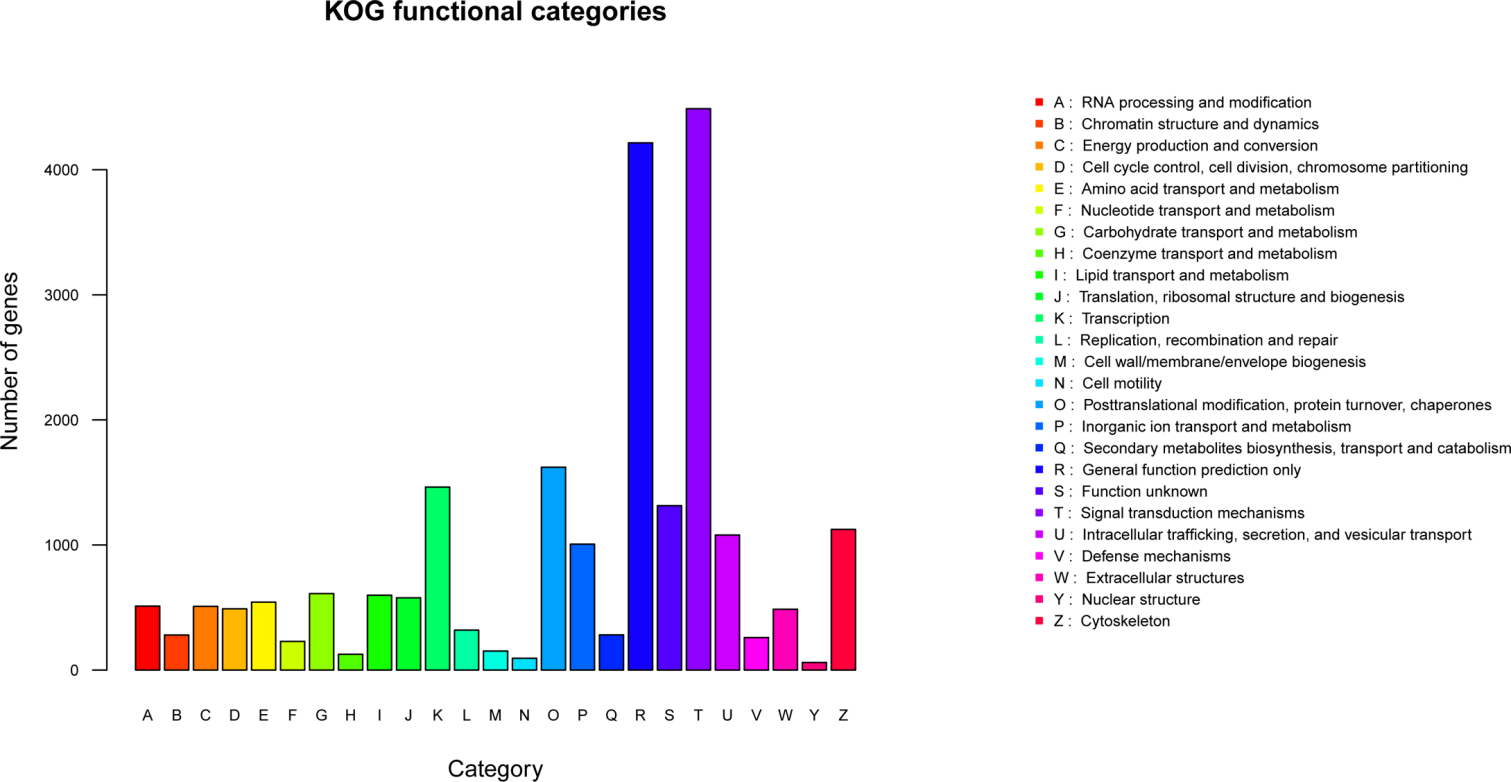
KOG classification of unigenes.

In all, 24,661 unigenes were annotated by the EuKaryotic Ortholog Groups (KOG) database and divided into 24 functional categories. These were mainly signal transduction mechanisms (20%) and general functions (18.76%; Fig 5).

**Fig 6:**
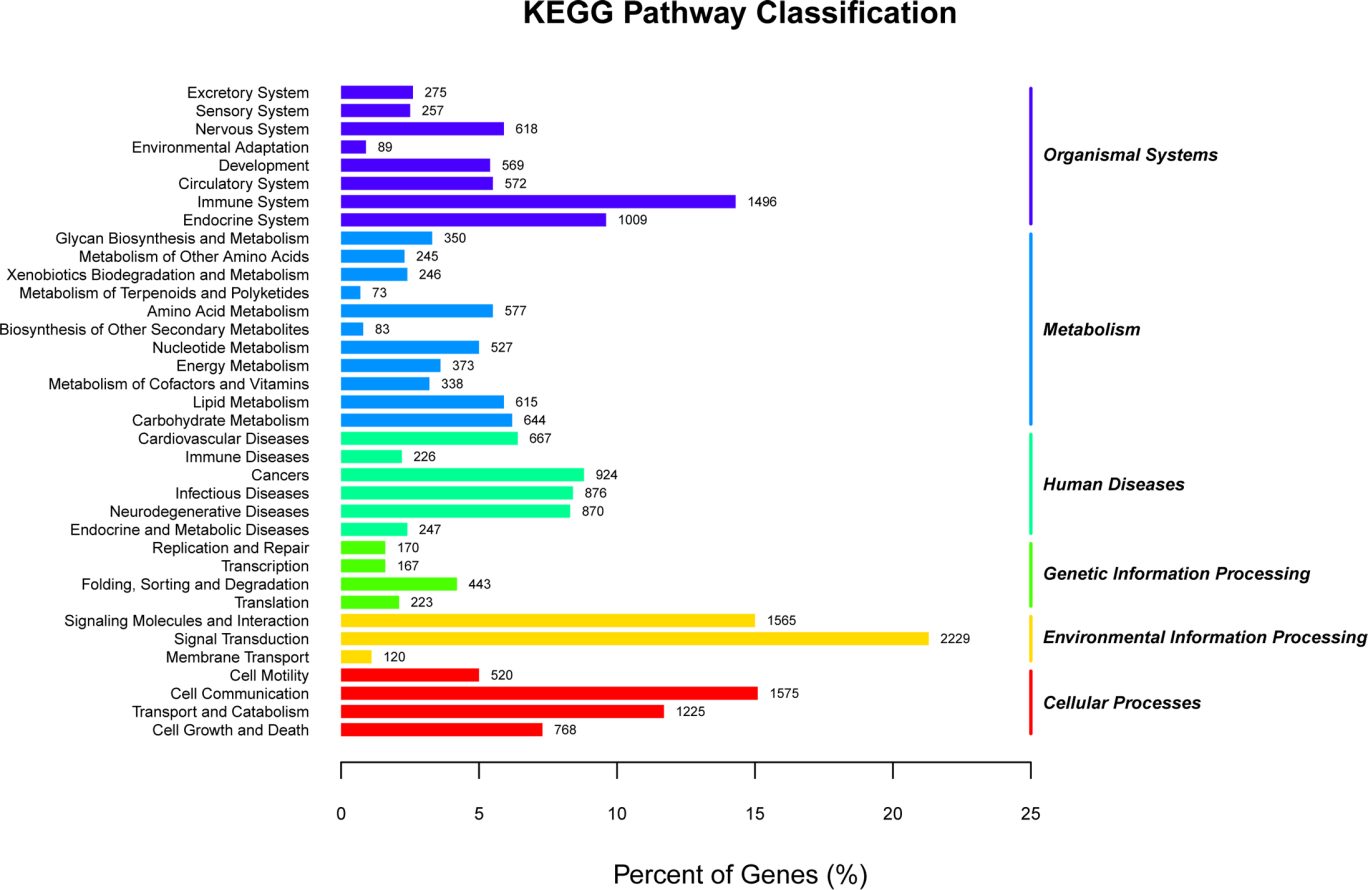
KEGG classification of unigenes.

In all, 18,525 unigenes were annotated by the Kyoto Encyclopedia of Genes and Genomes (KEGG) library into 262 pathways, covering metabolism, genetic information processing, environmental information processing, cellular process, human disease and six aspects of drug development (Fig 6).

### Identification of differentially expressed genes (DEGs)

**Fig 7:**
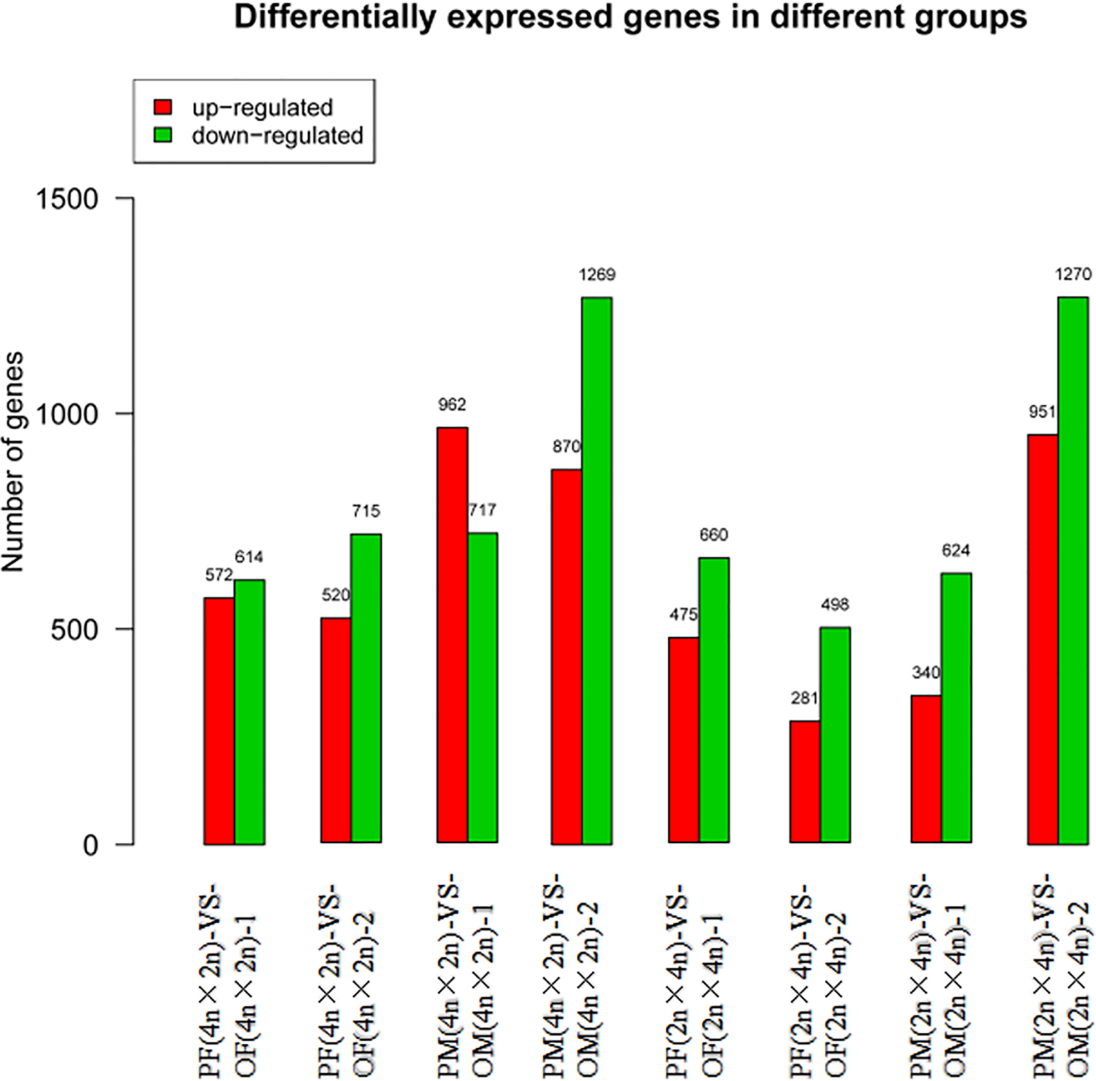
Statistically significant down regulated genes in the differentially. The red histogram in the figure represents the up regulated gene frequency, and the green histogram represents the down regulated gene frequency.

Significant DEGs were analyzed in each group. Most of the DEGs were found between diploid male parent and male offspring. The expression levels of *FMN2*, *Foxm1*, *Sox30* and other genes were up-regulated in male offspring, whereas the expression levels of *CYP17A1*, *CYP11A1*, *GNAQ* and other genes were down-regulated in male offspring. The fewest DEGs were present between diploid female parent and female offspring, where the expression levels of *MLL2*, *Ccnb1*, *IGF2*, and other genes were up-regulated in female offspring, and the expression levels of *HMR-1*, *Cyp27b1*, *CYP2J2* and other genes were down-regulated in female offspring (Fig 7).

**Fig 8:**
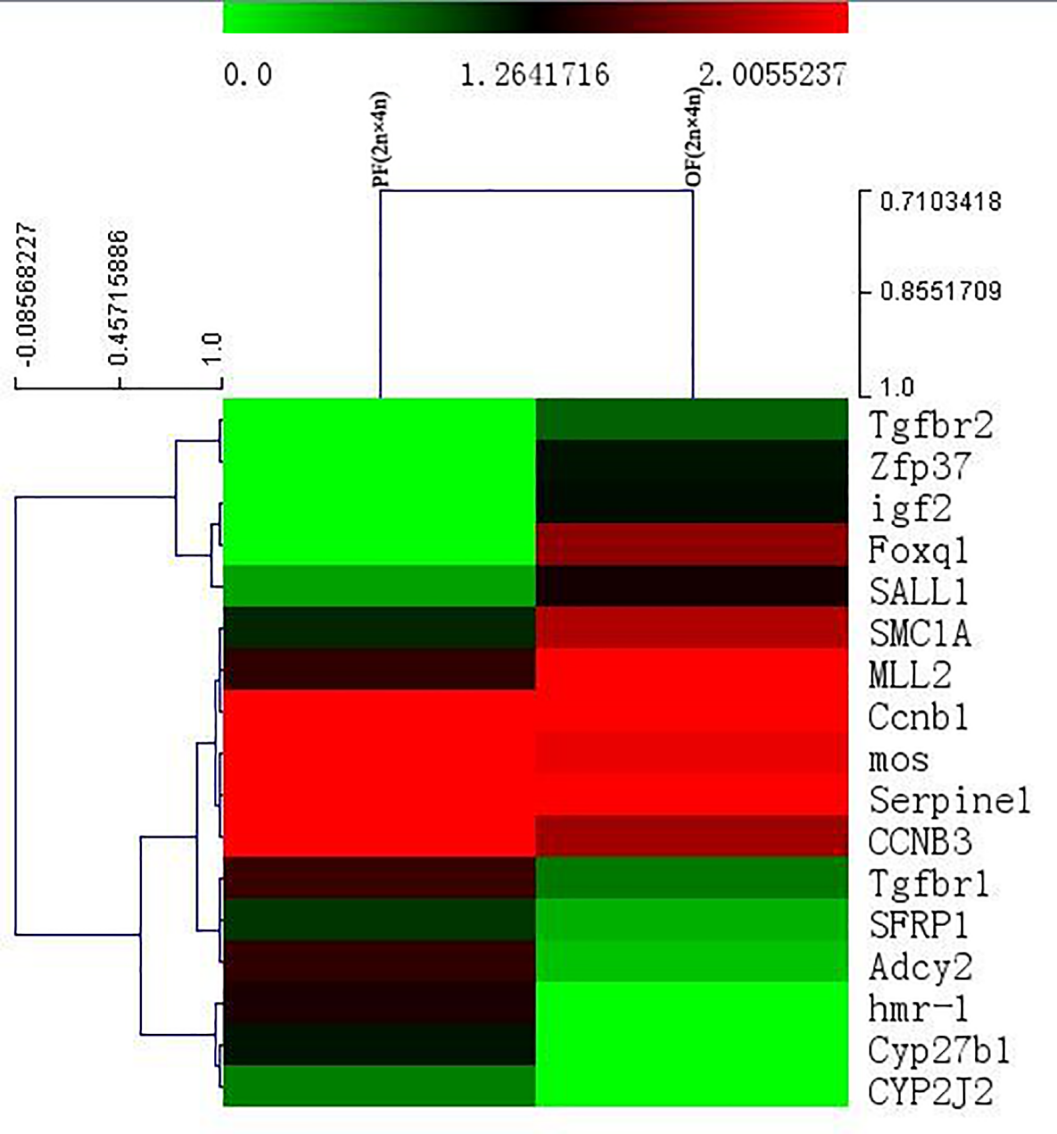
Fertility-related differentially expressed genes (DEGs) clustering analysis heatmap of PF(2n×4n)-VS-OF(2n×4n).

By screening for fertility-related DEGs, a total of 17 genes were firstly obtained from PF (2n × 4n)-VS-OF (2n × 4n), of which 8 genes were up-regulated in the progeny and 9 genes were down-regulated (S3 Table). The production of differential gene expression heat maps can give better understanding of the differences between gene distribution patterns. The expression levels of embryonic development-related (e.g., Foxq1) and DNA repair-related genes (e.g., SMC1A) were up-regulated in the progeny significantly. However, the expression levels of embryonic development-associated (e.g., hmr-1) and membrane-associated genes (e.g., Adcy2) were down-regulated in the progeny significantly (Fig 8).

**Fig 9:**
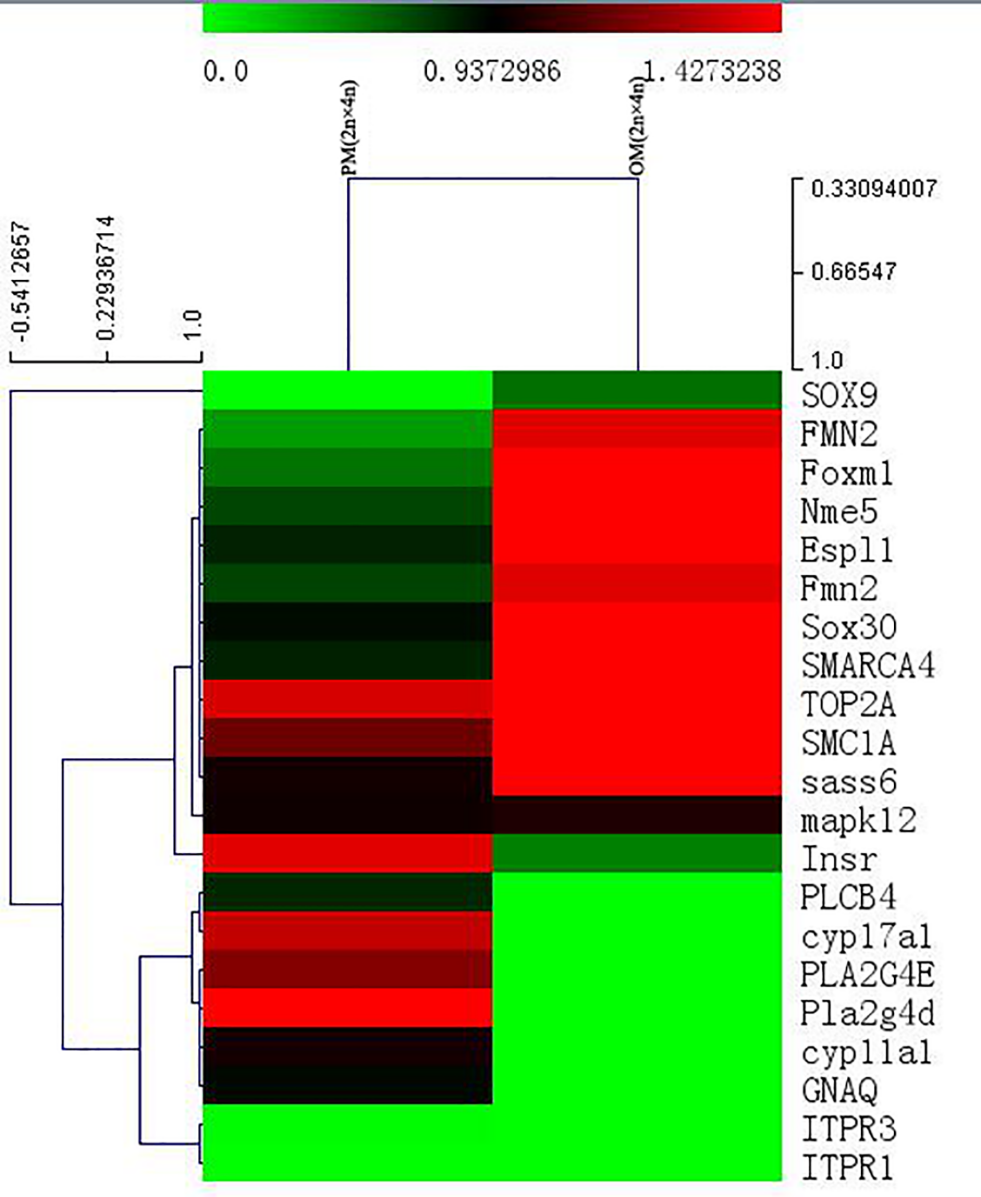
Fertility-related differentially expressed genes (DEGs) clustering analysis heatmap of PM(2n×4n)-VS-OM(2n×4n).

A total of 20 fertility-related *DEGs* were obtained from PM (2n × 4n)-VS-OM (2n × 4n), of which 10 genes were up-regulated in the progeny and 10 genes were down-regulated (S4 Table). The expression level of meiosis-related (e.g., *FMN2*) and transcription factor-related genes (e.g., *SOX9*) were up-regulated in the progeny significantly. However, the expression levels of steroid biosynthesis-related genes (e.g., *cyp17a* and *cyp11a1*) were down-regulated in the progeny significantly (Fig 9).

**Fig 10:**
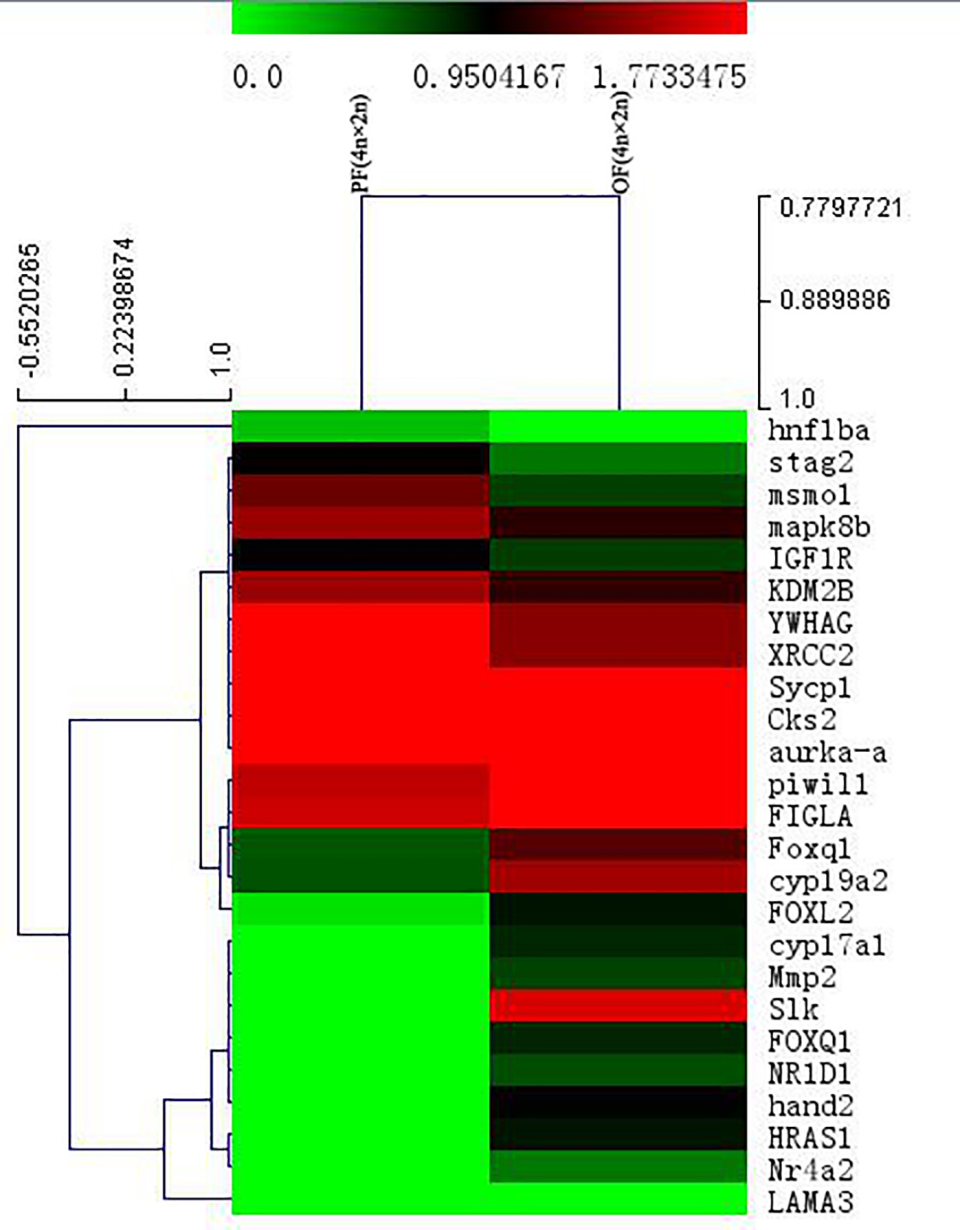
Fertility-related differentially expressed genes (DEGs) clustering analysis heatmap of PF(4n×2n)-VS-OF(4n×2n).

A total of 24 fertility-related *DEGs* were obtained from PF (4n × 2n)-VS-OF (4n × 2n), of which 13 genes were up-regulated in the progeny and 11 genes were down-regulated (S5 Table). The expression level of embryonic development-related (e.g., *LAMA3*) and transcriptional regulation-related genes (e.g., *hand2*) were up-regulated in the progeny significantly. However, the expression levels of mitosis-related genes (e.g., *Sycp1* and *stag2*) were down-regulated in the progeny significantly (Fig 10).

**Fig 11:**
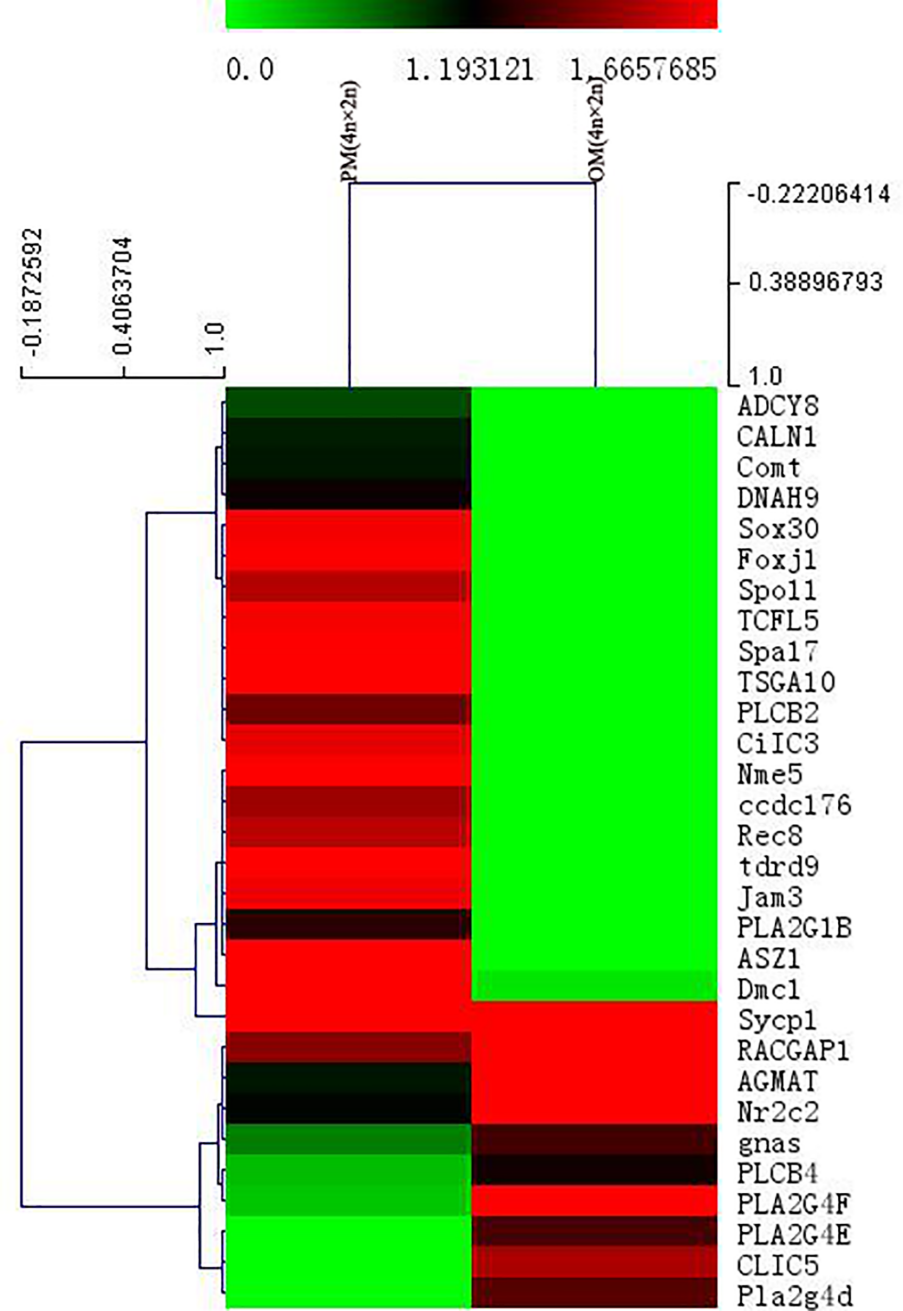
Fertility-related differentially expressed genes (DEGs) clustering analysis heatmap of PM(4n×2n)-VS-OM(4n×2n).

A total of 30 fertility-related DEGs were obtained from PM (4n × 2n)-VS-OM (4n × 2n), of which 9 genes were up-regulated in the progeny and 21 genes were down-regulated (S6 Table). The expression level of reverse transcription (*gnas*) and protein coding genes (e.g., *Pla2g4d*) were up-regulated in the progeny significantly. However, the expression levels of embryonic development related (e.g., *Sox30*) and sperm structure-related genes (e.g., *TSGA10*) were down-regulated in the progeny significantly (Fig 11).

### Protein-protein interaction (PPI) network analyses of the fertility-related DEGs

The protein-protein interaction network analyses of the fertility-related DEGs were performed. Of the fertility-related DEGs in the comparison of direct cross (2n × 4n) offspring and their parents, 27 genes were found to be linked to each other (S3 Fig). Of the fertility-related DEGs in the comparison of reciprocal cross (4n × 2n) offspring and their parents, 32 genes were linked to each other (S4 Fig). As a whole, 54 genes involved in poor fertility of hybrid triploid loach (direct or reciprocal crosses) were finally mined and 5 genes were coexisted in the two networks, namely *PLCB4*, *PLA2G4E*, *Pla2g4d*, *Nme5* and *cyp17a1*.

### Validation of DEGs by quantitative real-time polymerase chain reaction (qPCR) amplification

**Fig 12:**
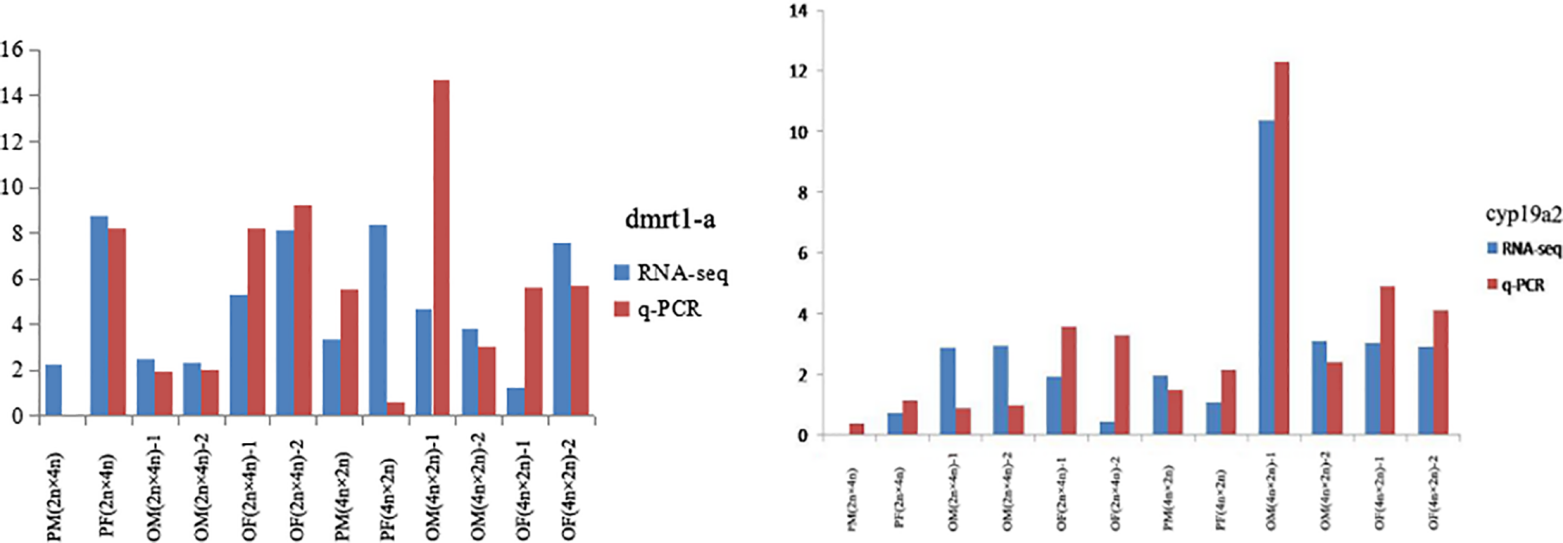
Comparison between RNA-seq and q-PCR result of Gene *dmrt1-a* and *cyp19a2*.

The expression levels of genes obtained using qPCR were compared with those obtained by RNA-seq to verify the accuracy of the latter approach. Fig 12 shows a comparison of qPCR and RNA-seq results for *dmrt1-a* and *cyp19a2*. It confirmed that the results of qPCR were consistent with those of the RNA-seq analysis, showing the reliability and accuracy of our transcriptome sequence expression analysis.

## Discussion

Triploid fish have the characteristics of fast growth, good meat quality, strong disease resistance, but poor fertility, so its breeding is of great significance [26]. These breeding characteristics have been receiving increasing attention in fishery resource management. It is generally accepted that triploid fish—being infertile—can transform the energy used in gonadal development into muscle growth, thus giving them a potential growth advantage. In addition, the sterility of triploid fish is of great significance for controlling the over-growth of fish and protecting natural germplasm resources. Moreover, triploid fish can also be used as ideal vectors for generating transgenic lines to address ecological safety problems and ethical concerns [27]. Most artificially induced triploid fish proved to be sterile [28–39]. However, there have been differences between reports. Thus, Yang [40] found that male triploid rainbow trout can produce spermatozoa, but a large number of malformed embryos appeared after hybridization, and all the progeny died during maturation. Yin et al. [41] found that the gonadal functions of triploid catfish differ between genders, as there was no difference between the normal and diploid testis, but the female ovary showed arrest of egg development at the oogonium stage. Arai [42] studied direct and reciprocal hybridization by using tetraploid loaches and diploid loaches of unknown origin. The male hybrids were sterile but female hybrids were fertile, and produced large triploid eggs (3n), and small haploid eggs (1n). Both types of egg could be fertilized and produce viable offspring. In the present study, hybrid triploid offspring were obtained by hybridization between tetraploid and diploid loaches, which are unique to China. Histology of the testes showed that spermatogonia, spermatocytes and large numbers of mature spermatozoa could be observed in diploid and triploid testes, but that the mature sperm content was lower than in normal diploid fish. The diploid and triploid female ovaries also contained second, third, and fourth phase oocytes, yolk granules and follicular membranes. However, the molecular mechanisms controlling fertility in such triploid fish have not been reported so far.

Transcriptomes are collections of all the RNAs of a particular tissue or cell that are transcribed at given developmental stages or functional state, including mRNA-encoding proteins and various noncoding RNAs, such as rRNAs, tRNAs, snoRNAs, snRNAs, and microRNAs. However, transcriptomal studies are typically restricted to mRNAs, and are important for studying the phenotypes and functions of cells. Transcriptomics is the study of gene transcription and transcriptional regulation in cells at the RNA level. Currently, high-throughput sequencing techniques have been used widely in transcriptomal studies of plants and animals, and many studies have also been carried out on the transcriptomes of fish, such as *Oncorhynchus mykiss* [43], *Lampetra japonica* [44], *Anguilla anguilla* [45], *Scophthalmus maximus* [46], and *Huso dauricus* [47]. Here we used high-throughput sequencing to study the transcriptomes of gonads from the offspring and parents of direct and reciprocal hybrids produced by crossing between diploid and tetraploid loaches. A total of 62,821 unigenes was obtained. Gene function annotations, classifications and metabolic pathways were analyzed, and sequence information of gonadal development-related genes in hybrid triploid loaches were obtained. Through screened differentially expressed genes related to fertility between parents and offspring and these genes were used to map protein-protein interaction networks, we found that there were 5 important genes, namely *PLCB4*, *PLA2G4E*, *Pla2g4d*, *Nme5* and *cyp17a1*, were up/down regulated in the offspring of whatever direct or reciprocal crosses. These genes participated in the estrogen signaling pathway and progesterone-mediated oocyte maturation. In 2015, Jiang [48] studied the proteomics and carried out transcriptomal analysis of homozygous male sterile individuals of the double haploid *Paralichthys olivaceus*, and screened the levels of *CYP11A1*, *CYP11B2*, *CYP17A1* and other genes. These genes play important roles in regulating the synthesis of sterol hormones and show low expression levels in infertile flounders. This suggests that infertility might be linked to low levels of hormones, similar to the differences between male and female offspring of diploid parents in this study. Jia et al. [49] reported that *Nme5* was expressed in pachytene primary spermatocytes, round spermatids and elongated spermatozoa, and participated in the whole process from meiosis to sperm deformation, and played an important role in spermatogenesis. *PLCB4* is one of the subtypes of phospholipase C (PLC), which controls cellular processes including neural signaling, cell growth and synaptic plasticity [50]. The *Pla2g4d* gene can directly regulate ovarian follicle development, or indirectly influence leptin secretion involved in the regulation of the sheep estrous cycle when cycles are initiated at the end of the anestrous season [51]. In addition, we also found that some genes were specific to the offspring and parents. For example, the *LACTBL1*, *SGK1*, *XLRS1*, *acbC*, and *SGK1* genes were expressed only in the offspring, in contrast, *Chst7*, *Lemd3*, *CMAS*, *SNRPB2*, and *KLHL24* genes were only expressed in the parents, so these genes might influence the differences in fertility.

In conclusion, the main reason for the poor fertility of hybrid triploid loach is probably because of changes in the expression levels of genes that regulate sex hormone levels and hormone response. The expression of genes related to gonad development of triploid hybrid parental was systematically analyzed by transcriptome, to analyze the reason of its occurrence in the view of molecular biology, and provides a theoretical basis for future research.

## Supporting information

S1 Table Quality test results of RNA. (DOCX)

S2 Table Sequencing results statistics. (DOCX)

S3 Table Table of PF(2n×4n)-VS-OF(2n×4n) of fertility-related gene. (DOCX)

S4 Table Table of PM(2n×4n)-VS-OM(2n×4n) of fertility-related gene. (DOCX)

S5 Table Table of PF(4n×2n)-VS-OF(4n×2n) of fertility-related gene. (DOCX)

S6 Table Table of PM(4n×2n)-VS-OM(4n×2n) of fertility-related gene. (DOCX)

S1 Fig Splice length distribution of unigenes and transcripts. (TIF)

S2 Fig GC content of unigenes and transcripts. (TIF)

S3 Fig Protein-protein interaction network for the enzymes encoded by the fertility-related DEGs in the comparsion of direct cross (2n × 4n) offspring and their parents. Different nodes represented different enzymes. The interactions among these enzymes were represented by different colorful lines.
(TIF)

S4 Fig Protein-protein interaction network for the enzymes encoded by the fertility-related DEGs in the comparsion of reciprocal cross (4n × 2n) offspring and their parents. Different nodes represented different enzymes. The interactions among these enzymes were represented by different colorful lines.
(TIF)
