# Supplementary material for: Gonadal transcriptome analysis of hybrid triploid loaches (Misgurnus anguillicaudatus) and their diploid and tetraploid parents
Source: PLoS One. 2018 May 24;13(5):e0198179. doi: 10.1371/journal.pone.0198179 (PMC5967825; doi:10.1371/journal.pone.0198179)
Supplement: S1 Table — (DOCX) [file pone.0198179.s001.docx]

| **Sample ID** | **O.D.**  **260/280** | **Amount**  **(µg)** | **rRNA**  **28S/18S** | **RIN** | **QC**  **Evaluation** |
| --- | --- | --- | --- | --- | --- |
| **PF(2n×4n)** | 2.09 | 14.28 | 1.1 | 8.3 | A |
| **PM(2n×4n)** | 2.07 | 5 | 1.2 | 8.2 | A |
| **OM(2n×4n)-1** | 2.16 | 20.64 | 1.5 | 8.9 | A |
| **OM(2n×4n)-2** | 2.06 | 19.59 | 1.7 | 9.3 | A |
| **OF(2n×4n)-1** | 2.13 | 29.57 | 1.5 | 8.7 | A |
| **OF(2n×4n)-2** | 2.14 | 5.81 | 1.7 | 9.2 | A |
| **OF(4n×2n)-1** | 2.11 | 17.61 | 1.6 | 9 | A |
| **OF(4n×2n)-2** | 2.15 | 25.57 | 1.5 | 8.7 | A |
| **PF(4n×2n)** | 2.13 | 38.35 | 1.6 | 7.6 | A |
| **PM(4n×2n)** | 1.89 | 1.05 | 1.2 | 8.1 | A |
| **OM(4n×2n)-1** | 2.06 | 6.72 | 1.4 | 9 | A |
| **OM(4n×2n)-2** | 2.08 | 15.86 | 1.5 | 8.8 | A |

**S1 Table. Quality test results of RNA**
