# Supplementary material for: Gonadal transcriptome analysis of hybrid triploid loaches (Misgurnus anguillicaudatus) and their diploid and tetraploid parents
Source: PLoS One. 2018 May 24;13(5):e0198179. doi: 10.1371/journal.pone.0198179 (PMC5967825; doi:10.1371/journal.pone.0198179)
Supplement: S2 Table — (DOCX) [file pone.0198179.s002.docx]

**S2 Table. Sequencing results statistics**

| **Sample** | **Raw Data** | | **Valid Data** | | **Valid%** | **Q20%** | **Q30%** | **GC%** |
| --- | --- | --- | --- | --- | --- | --- | --- | --- |
|  | **Read** | **Base** | **Read** | **Base** |  |  |  |  |
| **PF(2n×4n)** | 51402056 | 5.14G | 50869576 | 5.09G | 98.96 | 96.36 | 92.76 | 46.49 |
| **PM(2n×4n)** | 43464608 | 4.35G | 43014880 | 4.30G | 98.97 | 96.38 | 92.79 | 45.42 |
| **OF(2n×4n)-1** | 41847570 | 4.18G | 41441370 | 4.14G | 99.03 | 96.16 | 92.44 | 46.92 |
| **OF(2n×4n)-2** | 41161426 | 4.12G | 40820830 | 4.08G | 99.17 | 96.49 | 92.98 | 46.82 |
| **OM(2n×4n)-1** | 70406528 | 7.04G | 69537644 | 6.95G | 98.77 | 97.49 | 94.65 | 47.67 |
| **OM(2n×4n)-2** | 77598528 | 7.76G | 77105262 | 7.71G | 99.36 | 98.14 | 95.59 | 46.98 |
| **PF(4n×2n)** | 41211316 | 4.12G | 40797914 | 4.08G | 99.00 | 95.99 | 92.19 | 46.61 |
| **PM(4n×2n)** | 46209122 | 4.62G | 45746546 | 4.57G | 99.00 | 96.25 | 92.59 | 44.68 |
| **OF(4n×2n)-1** | 43100252 | 4.31G | 42697432 | 4.27G | 99.07 | 96.14 | 92.35 | 46.82 |
| **OF(4n×2n)-2** | 43896950 | 4.39G | 43470320 | 4.35G | 99.03 | 96.39 | 92.79 | 46.80 |
| **OM(4n×2n)-1** | 70161008 | 7.02G | 69568122 | 6.96G | 99.15 | 98.06 | 95.41 | 45.97 |
| **OM(4n×2n)-2** | 90883062 | 9.09G | 90039262 | 9.00G | 99.07 | 97.84 | 95.02 | 46.38 |
