# Supplementary material for: Gonadal transcriptome analysis of hybrid triploid loaches (Misgurnus anguillicaudatus) and their diploid and tetraploid parents
Source: PLoS One. 2018 May 24;13(5):e0198179. doi: 10.1371/journal.pone.0198179 (PMC5967825; doi:10.1371/journal.pone.0198179)
Supplement: S3 Table — (DOCX) [file pone.0198179.s003.docx]

**S3 Table. Table of PF(2n×4n)-VS-OF(2n×4n) of fertility-related gene**

| **gene-ID** | **name** | **annotation** | **log_2_fold_change** | **regulation** |
| --- | --- | --- | --- | --- |
| comp176170_c0 | Foxq1 | forkhead box protein Q | -5.28 | up |
| comp196114_c0 | Tgfbr1 | TGF-beta receptor type-1 | 2.80 | down |
| comp196343_c0 | Tgfbr2 | TGF-beta receptor type-2 | -4.00 | up |
| comp184856_c0 | Serpine1 | plasminogen activator inhibitor-1 | 1.93 | down |
| comp172303_c0 | MLL2 | myeloid/lymphoid or mixed-lineage leukemia protein 2 | -2.12 | up |
| comp199334_c0 | SMC1A | centromeric protein E | -2.02 | up |
| comp197141_c0 | hmr-1 | cadherin EGF LAG seven-pass G-type receptor 1 | 4.84 | down |
| comp180134_c0 | Adcy2 | adenylate cyclase 2 | 4.00 | down |
| comp185438_c0 | igf2 | insulin-like growth factor 1 | -3.76 | up |
| comp189750_c0 | CCNB3 | cyclin B | 2.04 | down |
| comp181085_c0 | Ccnb1 | cyclin B | -1.58 | up |
| comp179226_c0 | mos | proto-oncogene serine/threonine-protein kinase mos | 1.82 | down |
| comp187183_c0 | SFRP1 | secreted frizzled-related protein 1 | 3.29 | down |
| comp193310_c0 | SALL1 | KRAB domain-containing zinc finger protein | -2.55 | up |
| comp174810_c0 | Zfp37 | KRAB domain-containing zinc finger protein | -5.52 | up |
| comp187247_c0 | Cyp27b1 | cytochrome P450, family 27 | 6.05 | down |
| comp190931_c0 | CYP2J2 | cytochrome P450, family 2 | 3.86 | down |
