# Supplementary material for: Gonadal transcriptome analysis of hybrid triploid loaches (Misgurnus anguillicaudatus) and their diploid and tetraploid parents
Source: PLoS One. 2018 May 24;13(5):e0198179. doi: 10.1371/journal.pone.0198179 (PMC5967825; doi:10.1371/journal.pone.0198179)
Supplement: S4 Table — (DOCX) [file pone.0198179.s004.docx]

**S4 Table. Table of PM(2n×4n)-VS-OM(2n×4n) of fertility-related gene**

| **gene_ID** | **name** | **annotation** | **log_2_fold_change** | **regulation** |
| --- | --- | --- | --- | --- |
| comp191872_c0 | cyp17a1 | cytochrome P450, family 17 | 5.38 | down |
| comp195515_c0 | cyp11a1 | cytochrome P450, family 11 | 5.60 | down |
| comp182917_c0 | Pla2g4d | phospholipase A2 | 7.83 | down |
| comp175151_c1 | PLA2G4E | phospholipase A2 | 5.63 | down |
| comp182691_c0 | Nme5 | nucleoside-diphosphate kinase | -3.39 | up |
| comp186829_c0 | Sox30 | transcription factor SOX, other | -3.02 | up |
| comp174599_c2 | SOX9 | transcription factor SOX7/8/9/10/18 | -5.04 | up |
| comp196550_c0 | sass6 | KRAB domain-containing zinc finger protein | -2.29 | up |
| comp197275_c0 | Foxm1 | forkhead box protein M | -4.62 | up |
| comp183773_c0 | Insr | insulin receptor | 2.65 | down |
| comp192786_c0 | FMN2 | formin 2 | -3.69 | up |
| comp199461_c0 | Espl1 | separase | -3.37 | up |
| comp197082_c0 | TOP2A | DNA topoisomerase II | -3.39 | up |
| comp199334_c0 | SMC1A | centromeric protein E | -2.90 | up |
| comp159382_c0 | GNAQ | guanine nucleotide binding protein | 6.27 | down |
| comp182547_c0 | mapk12 | p38 MAP kinase | 2.87 | down |
| comp185802_c0 | PLCB4 | phospholipase C, beta | 2.94 | down |
| comp176368_c0 | ITPR1 | inositol 1,4,5-triphosphate receptor | 4.29 | down |
| comp192297_c0 | ITPR3 | inositol 1,4,5-triphosphate receptor | 3.54 | down |
| comp183606_c1 | SMARCA4 | SWI/SNF-related matrix-associated actin-dependent regulator of chromatin subfamily A member 2/4 | -5.32 | up |
