# Supplementary material for: Gonadal transcriptome analysis of hybrid triploid loaches (Misgurnus anguillicaudatus) and their diploid and tetraploid parents
Source: PLoS One. 2018 May 24;13(5):e0198179. doi: 10.1371/journal.pone.0198179 (PMC5967825; doi:10.1371/journal.pone.0198179)
Supplement: S5 Table — (DOCX) [file pone.0198179.s005.docx]

**S5 Table. Table of PF(4n×2n)-VS-OF(4n×2n) of fertility-related gene**

| **gene_ID** | **name** | **annotation** | **log2fold_change** | **regulation** |
| --- | --- | --- | --- | --- |
| comp184446_c0 | HRAS1 | GTPase HRas | -5.49 | up |
| comp188464_c0 | mapk8b | c-Jun N-terminal kinase | 1.50 | down |
| comp197453_c1 | Sycp1 | non-specific serine/threonine protein kinase | 1.72 | down |
| comp191015_c0 | piwil1 | aubergine | -1.70 | up |
| comp178303_c0 | FOXQ1 | forkhead box protein Q | -3.40 | up |
| comp185596_c0 | cyp19a2 | cytochrome P450, family 19 | -2.64 | up |
| comp189415_c0 | cyp17a1 | cytochrome P450, family 17 | -3.03 | up |
| comp186271_c0 | msmo1 | methylsterol monooxygenase | 2.04 | down |
| comp197092_c0 | NR1D1 | nuclear receptor, subfamily 1, group D, member 1 | -2.81 | up |
| comp177127_c0 | Nr4a2 | nuclear receptor, subfamily 4, group A, member 2 | -3.68 | up |
| comp192808_c0 | aurka-a | aurora kinase A | 1.14 | down |
| comp185660_c0 | YWHAG | tyrosine 3-monooxygenase | 1.37 | down |
| comp198896_c0 | IGF1R | insulin-like growth factor 1 receptor | 1.60 | down |
| comp191798_c0 | Mmp2 | matrix metalloproteinase-9 (gelatinase B) | -2.71 | up |
| comp184970_c0 | FOXL2 | forkhead box protein L | -2.46 | up |
| comp180986_c1 | FIGLA | factor in the germline alpha | -1.84 | up |
| comp176083_c0 | hand2 | heart-and neural crest derivatives-expressed protein | -4.50 | up |
| comp197068_c0 | KDM2B | F-box and leucine-rich repeat protein 10 | 1.27 | down |
| comp185892_c0 | LAMA3 | laminin, alpha 3 | -3.91 | up |
| comp194852_c0 | hnf1ba | transcription factor 2 | 3.43 | down |
| comp192757_c0 | Slk | STE20-like kinase | -5.37 | up |
| comp195494_c0 | stag2 | cohesin complex subunit SA-1/2 | 1.63 | down |
| comp174079_c0 | XRCC2 | DNA-repair protein XRCC2 | 1.38 | down |
| comp181770_c0 | Cks2 | cyclin-dependent kinase regulatory subunit CKS1 | 1.11 | down |
