# Supplementary material for: Gonadal transcriptome analysis of hybrid triploid loaches (Misgurnus anguillicaudatus) and their diploid and tetraploid parents
Source: PLoS One. 2018 May 24;13(5):e0198179. doi: 10.1371/journal.pone.0198179 (PMC5967825; doi:10.1371/journal.pone.0198179)
Supplement: S6 Table — (DOCX) [file pone.0198179.s006.docx]

**S6 Table. Table of PM(4n×2n)-VS-OM(4n×2n) of fertility-related gene**

| **gene_ID** | **name** | **annotation** | **log2fold_change** | **regulation** |
| --- | --- | --- | --- | --- |
| comp186829_c0 | Sox30 | transcription factor SOX, other | 9.23 | down |
| comp182691_c0 | Nme5 | nucleoside-diphosphate kinase | 8.87 | down |
| comp192783_c0 | TSGA10 | myosin heavy chain | 9.30 | down |
| comp191848_c0 | Foxj1 | forkhead box protein J1 | 8.41 | down |
| comp189897_c0 | TCFL5 | transcription factor-like 5 | 8.16 | down |
| comp193983_c0 | Spa17 | microtubule-associated protein 1 | 7.91 | down |
| comp193070_c0 | Spo11 | meiotic recombination protein SPO11 | 7.70 | down |
| comp193716_c0 | CiIC3 | nucleoside-diphosphate kinase | 7.26 | down |
| comp198951_c0 | DNAH9 | adenosinetriphosphatase | 7.39 | down |
| comp186560_c0 | ASZ1 | ankyrin | 6.90 | down |
| comp197104_c0 | tdrd9 | adenosinetriphosphatase | 6.96 | down |
| comp191372_c0 | Rec8 | cohesin complex subunit SCC1 | 5.90 | down |
| comp181988_c0 | Dmc1 | meiotic recombination protein DMC1 | 6.40 | down |
| comp197518_c0 | Jam3 | junction adhesion molecule 3 | 5.72 | down |
| comp180416_c0 | CLIC5 | chloride intracellular channel 5 | -6.83 | up |
| comp186735_c0 | CALN1 | calmodulin | 8.04 | down |
| comp190743_c0 | ADCY8 | adenylate cyclase 8 | 6.88 | down |
| comp189643_c0 | ccdc176 | phospholipase C, beta | 6.04 | down |
| comp188749_c0 | PLCB2 | phospholipase C, beta | 5.91 | down |
| comp182917_c0 | Pla2g4d | phospholipase A2 | -6.02 | up |
| comp192464_c0 | PLA2G4F | phospholipase A2 | -4.08 | up |
| comp175151_c1 | PLA2G4E | phospholipase A2 | -5.20 | up |
| comp192206_c0 | gnas | guanine nucleotide binding protein | -2.67 | up |
| comp185802_c0 | PLCB4 | phospholipase C, beta | -3.36 | up |
| comp175908_c0 | PLA2G1B | phospholipase A2 | 5.40 | down |
| comp152275_c0 | Comt | catechol O-methyltransferase | 8.12 | down |
| comp197453_c1 | Sycp1 | non-specific serine | 5.08 | down |
| comp187985_c0 | Nr2c2 | nuclear receptor | -3.10 | up |
| comp197724_c0 | RACGAP1 | myosin IX | -2.53 | up |
| comp190744_c0 | AGMAT | agmatinase | -2.25 | up |
