# Supplementary figures and images for: Gonadal transcriptome analysis of hybrid triploid loaches (Misgurnus anguillicaudatus) and their diploid and tetraploid parents
Source: PLoS One. 2018 May 24;13(5):e0198179. doi: 10.1371/journal.pone.0198179 (PMC5967825; doi:10.1371/journal.pone.0198179)

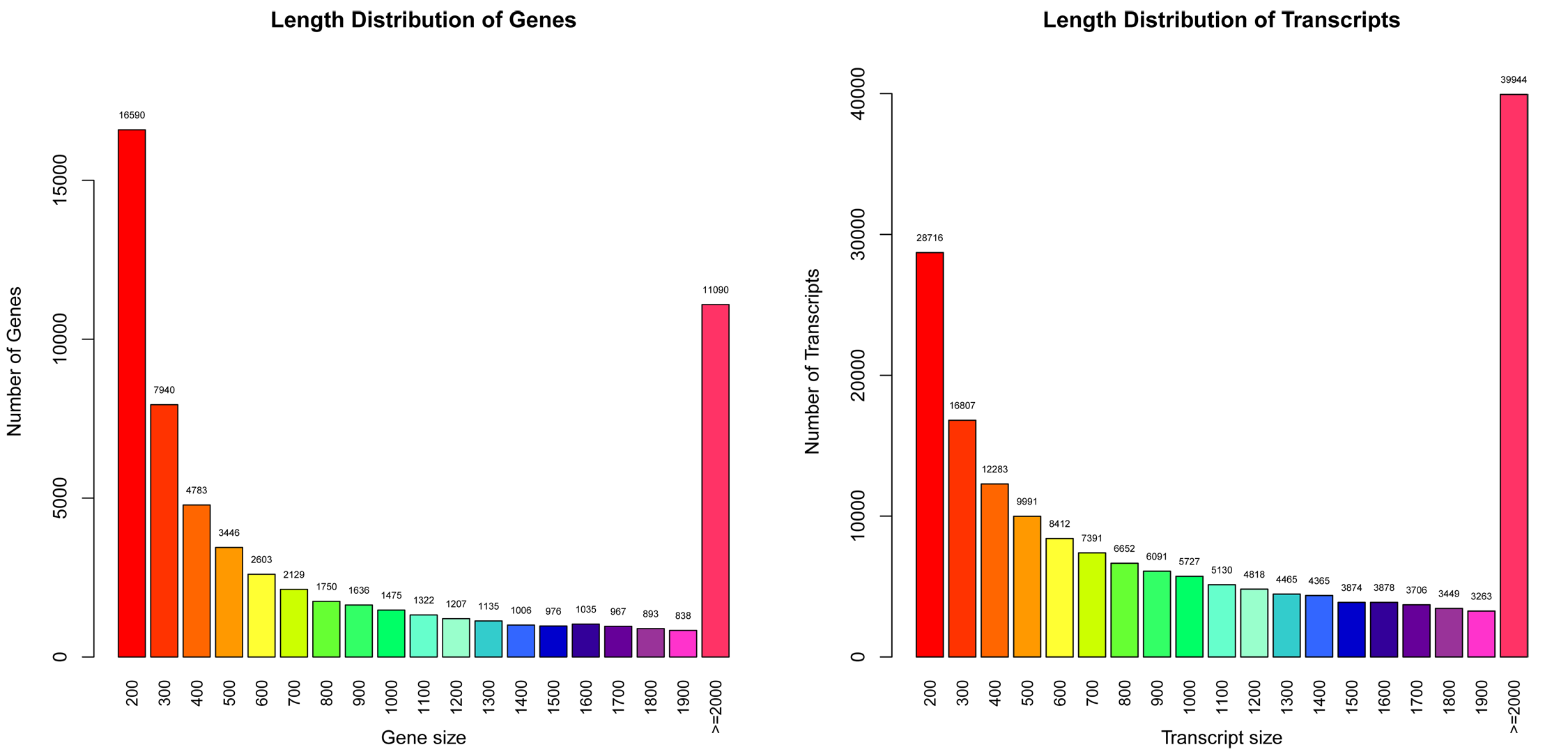

Supplement: S1 Fig — (TIF) [file pone.0198179.s007.tif]

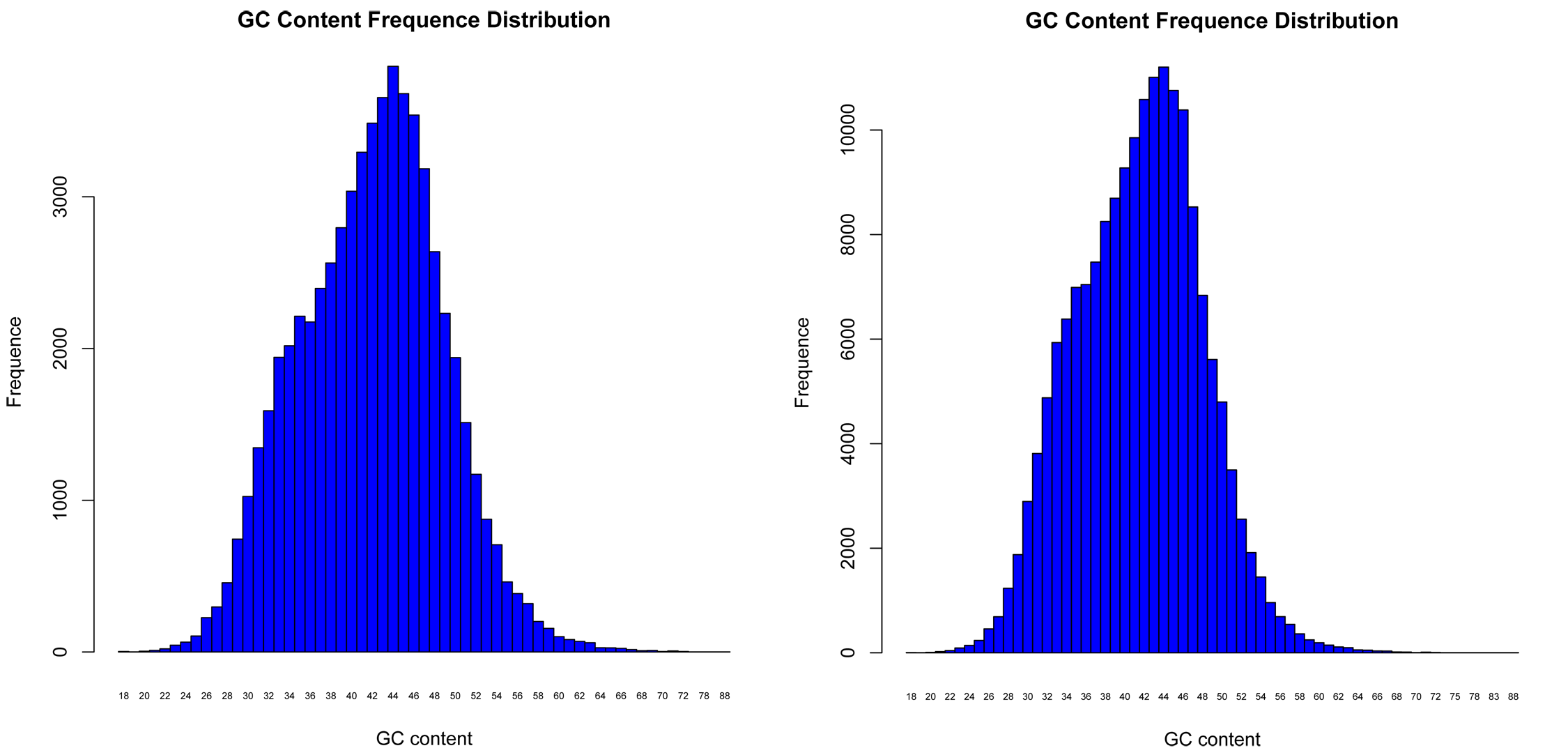

Supplement: S2 Fig — (TIF) [file pone.0198179.s008.tif]

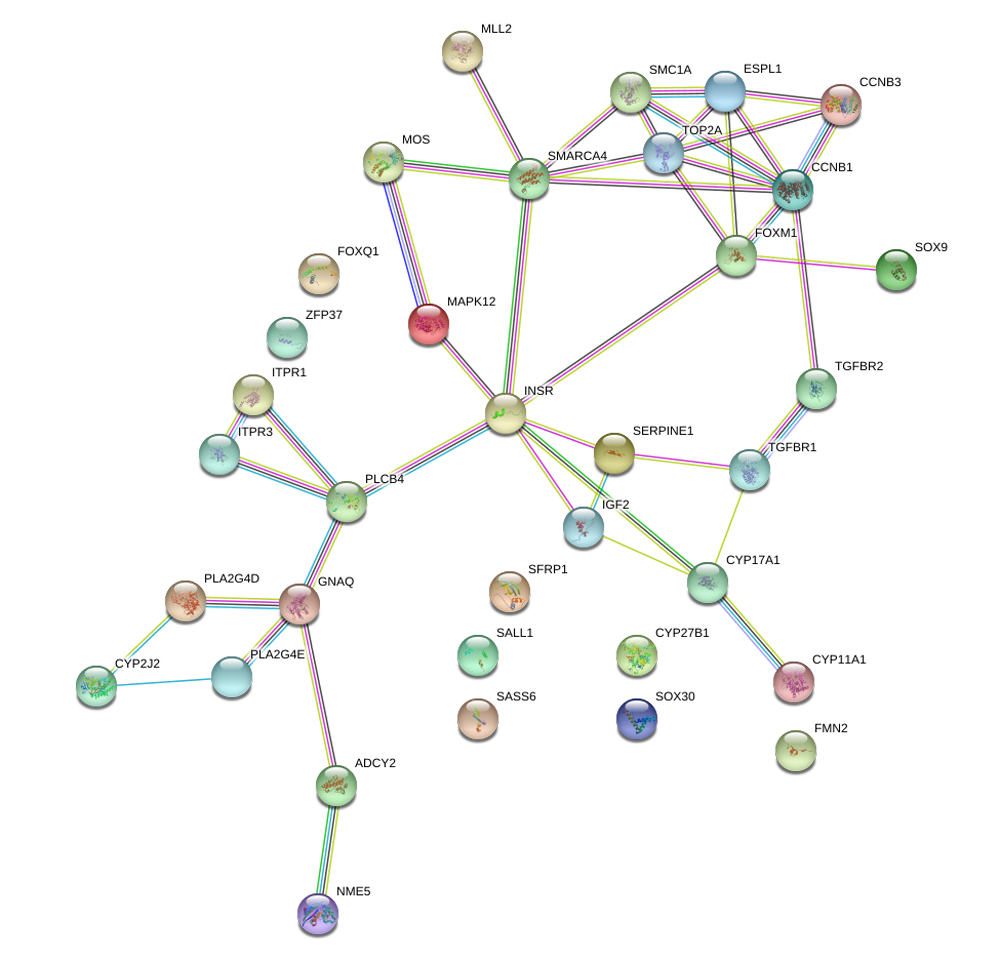

Supplement: S3 Fig — Different nodes represented different enzymes. The interactions among these enzymes were represented by different colorful lines. (TIF) [file pone.0198179.s009.tif]

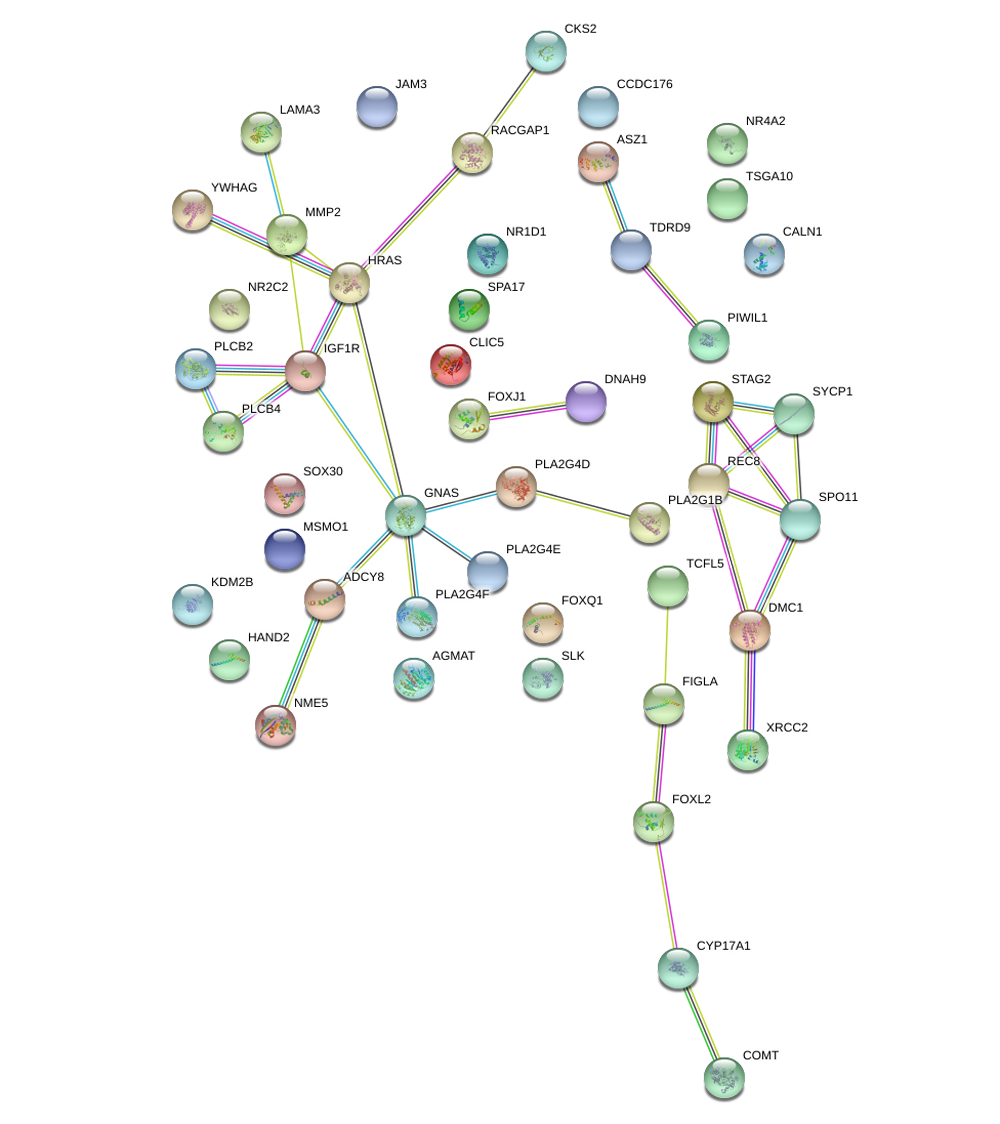

Supplement: S4 Fig — Different nodes represented different enzymes. The interactions among these enzymes were represented by different colorful lines. (TIF) [file pone.0198179.s010.tif]
